# Rumen Biogeographical Regions and Microbiome Variation

**DOI:** 10.3390/microorganisms11030747

**Published:** 2023-03-14

**Authors:** Macey P. Soltis, Sarah E. Moorey, Amanda M. Egert-McLean, Brynn H. Voy, Elizabeth A. Shepherd, Phillip R. Myer

**Affiliations:** Department of Animal Science, University of Tennessee, Knoxville, TN 37996, USA

**Keywords:** rumen, microbiome, rumen sacs

## Abstract

The rumen is a complex organ that is critical for its host to convert low-quality feedstuffs into energy. The conversion of lignocellulosic biomass to volatile fatty acids and other end products is primarily driven by the rumen microbiome and its interaction with the host. Importantly, the rumen is demarcated into five distinct rumen sacs as a result of anatomical structure, resulting in variable physiology among the sacs. However, rumen nutritional and microbiome studies have historically focused on the bulk content or fluids sampled from single regions within the rumen. Examining the rumen microbiome from only one or two biogeographical regions is likely not sufficient to provide a comprehensive analysis of the rumen microbiome and its fermentative capacity. Rumen biogeography, digesta fraction, and microbial rumen–tissue association all impact the diversity and function of the entirety of the rumen microbiome. Therefore, this review discusses the importance of the rumen biographical regions and their contribution to microbiome variation.

## 1. Introduction

The beef cattle industry is a segmented system beginning in the cow-calf sector, moving through a grazing/pre-conditioning program, and finally, to a feedlot before harvest. Cattle are foregut fermenters that utilize a synergistic relationship with their microbiome to produce meat and milk during their life cycle. Cattle possess the capability to break down recalcitrant plant material into useable energy, which is largely due to the diverse degrading enzymes encoded by the commensal and symbiotic microbes [1,2] and the distinct structure of the forestomach [3]. With the aid of commensal and symbiotic microbes, the non-glandular, non-secretory rumen [4] is continuously fermenting feedstuffs and is kept as an anaerobic chamber with an average pH of 6.5 [5]. However, these conditions are a gross assessment, as each of the five rumen sacs effectively produces further distinct environments. These five different rumen sacs create different niche environments, demarcating specialized internal framework [3,6,7]. The pillars, grooves, and folds increase the internal complexity of the rumen and therefore, how various microbial communities settle into these different ecosystems, impacting the diversity of microorganism populations inhabiting the rumen, including bacteria, archaea, protozoa, and fungi [8]. Microbial communities contribute not only to the nutrition of cattle but also to the health and growth of the animal throughout the production cycle. Over their lifetime, cattle will experience changes in diet, location, and management. However, cattle possess an innate adaptable microbiome that can acclimate to the many changes that will ensue [9]. This adaptable microbiome will guarantee essential microbes outcompete and thrive within the rumen. Commensal and symbiotic microbiota maintain essential roles within both cattle and throughout the production cycle, due to their importance in providing energy for the host to grow and efficiently generate commodities for human use. Therefore, it is critical to understand how localized biogeographical regions generated by rumen anatomy and physiology may affect the rumen microbiome. In the future, these data may provide approaches on how to better moderate some of the recognized consequences of the typical production cycle, such as ruminal acidosis or methane production.

## 2. Anatomy of the Rumen Affecting the Microbiome

**Figure 1:**
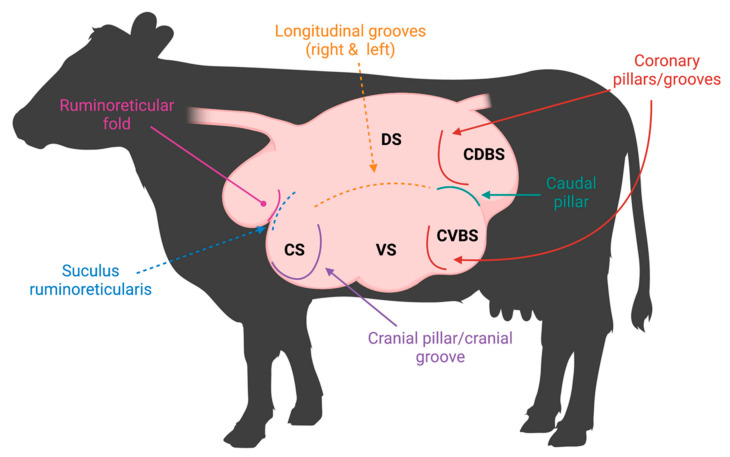
Rumen pillars and grooves visualized in a labeled rumen. Visual representation of musculature demarcating the rumen both internally (pillars) and externally (grooves). The rumen sacs are labeled as follows: cranial sac (CS), caudodorsal blind sac (CDBS), caudoventral blind sac (CVBS), dorsal sac (DS), and ventral sac (VS).

Cattle are essential to the global protein supply due to their unique ability to consume plant material that is indigestible to mammals and convert it into usable energy [10]. The fermentation of plant material is performed within cattle’s four-chambered stomach, which consists of the reticulum, rumen, omasum, and abomasum [10], where the rumen provides a suitable environment for microbes to reside. Importantly, due to pillars, grooves, and folds demarcating the rumen, there are five distinct rumen sacs, creating variable sub-ruminal environments (Figure 1). Therefore, these distinct sacs may contain microbial communities that have acclimated to the varying biogeographical regions within the rumen.

The anatomical configuration of the rumen creates functional variation within each rumen sac due to assorted accumulation of feed particles, saliva, and volatile fatty acids (VFAs). Investigation of different ruminal fermentation parameters, such as VFA concentration, pH, ammonia nitrogen (N), and other minerals (sodium, potassium, calcium, chloride, and phosphorus), were observed from different rumen sac sampling [11]. Cranial, dorsal, caudodorsal, caudoventral, and two different areas within the ventral sac were sampled to obtain a complete assessment of the ruminal environment. Differences were observed in fermentation parameters between the cranial sac and the other four sacs [11]. Notably, the cranial sac had greater pH and sodium concentrations, while also having lower total VFA concentrations [11], due to the increased amount of saliva and feed particles within the cranial sac. Although microbial communities were not investigated, microbial variation may occur, as favorable conditions for certain microbial species can be present [12]. Amylolytic bacteria favor and thrive in lower pH environments caused by high grain diets, while this environment will inhibit the growth of cellulolytic bacterial species [12]. Differences observed in rumen fermentation parameters are due to sample site [11], which can be attributed to anatomical distinctions made among the ruminal cavity. Pillars, grooves, and folds form the anatomical distinctions and thus, five separate rumen sacs. Environmental conditions of the rumen sacs continuously fluctuate due to ruminal contractions, stratification, and digestibility of feedstuffs occurring throughout beef cattle production. Fluctuations in each ruminal sac environment may instigate ruminal dysbiosis causing adjustments to microbial communities. Improved understanding of the five ruminal sacs, their distinct microbial communities, and the interactions between host and microbiome will reduce undesired consequences caused by unavoidable environmental, diet, and management changes occurring during production.

### 2.1. Ruminal Environment Variations and Their Impact on Microbial Community Variation

The five rumen sacs establish unique conditions that favor specific microbial species, and therefore, these microbes may occupy specific sacs with less competition [3,13] For example, in lambs, epimural bacteria appear to be less numerous in the caudal sac than other sacs of the rumen; yet, in the adult animal, the microbial colonization was greater in the dorsal sac than the ventral [13]. These differences in epimural microbial populations highlight the variation attributed to age and age × diet interactions that may occur due to these ruminal compartments or even colonization potential. Indeed, it has been shown that some epithelium-associated microbes adhere directly to the epithelium or directly to the glycocalyx of the first colonizing strains [13,14,15]. These interactions vary based on the location within the rumen and available substrates. Ruminal chemistry can also vary, as significant differences in VFA concentrations were observed when five different positions—dorsal, middle, ventral, reticulum, and dorsal posterior regions—were sampled within the reticulo-rumen [16]. However, pH was only different between ventral and dorsal regions, with an average of 0.43 pH less in the dorsal region compared to the ventral [16]. Sampling time was possibly a confounding factor among these parameters, as the dorsal region consistently contained greater VFA concentrations and was 38% greater than the ventral region [16]. This interaction between the host environmental conditions and the microbial communities is critical to appreciating the ruminal niches and how they establish.

**Figure 2:**
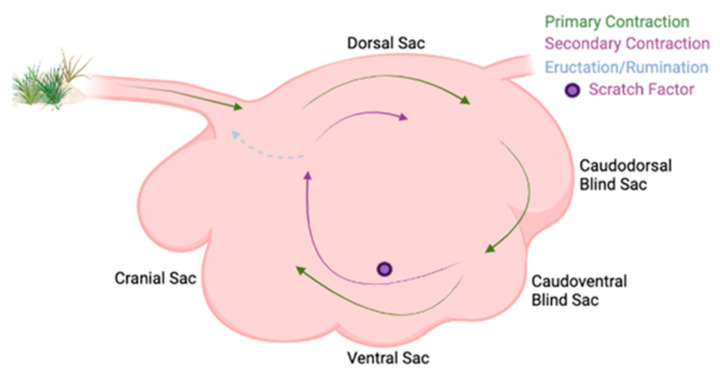
Ruminal contraction cycles in a labeled rumen. Labeled rumen sacs with ruminal contractions shown. The primary contraction cycle is shown with the green arrows in the clockwise cycle and it follows within the rumen. The secondary contraction cycle is shown with the purple arrows and is not explicitly explained in the text, but is where eructation, rumination, and continual fermentation occur, and the feedstuffs are not passed onto the omasum.

Beyond differences in ruminal sac chemistry, stratification of rumen digesta occurs based on specific gravity differentiating rumen liquid from rumen solid digesta. The liquid layer is topped by an accumulation of low-density forage particles that form the fiber mat [7]. Ruminal stratification is best demonstrated when cattle are fed high forage diets [17] due to slower rate of fermentation and greater abundance of low specific gravity feedstuffs that rise above the rumen liquid and create the fiber mat. Vertical stratification of rumen contents can further be explained by the primary contraction cycle that facilitates the flow of denser feedstuffs to the ventral sac, where liquid digesta accumulates (Figure 2) [18]. Less dense plant materials are not cycled during primary contractions, and thus, gather atop the liquid layer forming the fiber mat in the dorsal sac [19]. Due to the primary contraction cycle enabling the accumulation of low-density plant feedstuffs in the dorsal sac, dorsal locations are more likely to be affected by stratified rumen contents [20]. When examining whether ruminal bacterial structure, population, and fermentation parameters differed between ruminal locations, rumen digesta samples were obtained from the cranial ventral, cranial dorsal, central rumen, caudal dorsal, and caudal ventral sacs [21]. No differences were observed between bacterial structure and sampling location; however, sampling was limited to the ventral and dorsal sacs. Greater pH was detected in the cranial regions [21], which influences microbial growth and is an important fermentation variable, although differences were lost due to the analysis technique [21,22]. Polymerase chain reaction–denaturing gradient gel electrophoresis (PCR-DGGE) was used to discern bacterial communities but is unable to assess bacterial species as in-depth as high-throughput sequencing (HTS). Therefore, the use of HTS is ideal to detect minor bacterial species variation between rumen sacs. Although no significant differences were observed between rumen sacs, eight specific bacterial species increased in caudal regions. Ruminal contractions impact the movement and stratification of feedstuffs and therefore microbial species which may have affected the greater populations seen among caudal regions and more basic pH among the cranial regions.

Considering the primary, secondary, and extraruminal contractions, rumen motility is an important factor contributing to microbial variation in the rumen [23]. Rumen motility permits the continuous colonization and interactions with ingesta, resulting in efficient fermentation processes [24,25]. Arowolo et al. examined the effect of motility in vitro using the RUSITEC (rumen simulation technique) system, hypothesizing that changes in rotation speeds would impact rumen fermentation, saturation of dissolved gases, hydrogen, and methane emissions, microbial protein synthesis, and selected microbial populations. A moderate in vitro rotation speed (15 rpm) resulted in the greatest total VFA and microbial protein concentrations, while also increasing fungi and protozoa counts in the solid contents and bacteria and fungi in the liquid contents [23]. This work was important in providing insights into the importance of motility on rumen function, fermentation, and microbial activity. In vivo, motility and the frequency of primary contractions are important in the mixing and passage of ruminal content to the omasum, which has been shown to be reduced with episodes of ruminal dysfunction, such as ruminal acidosis [26]. The change in microbiome and microbial fermentation as a result of acidosis and motility may affect VFA flow to the ventral sac, subsequently reducing the absorption of VFA from the rumen [27]. Yet, rumen motility is affected by numerous factors, such as blood flow, diet, nervous system, and rumen dysfunction [10]. In the southeastern US, where fescue toxicosis is a primary concern for beef producers, ergovaline has been shown to impact rumen motility through a decrease in frequency and amplitude of ruminal contractions, while also impacting rumen fill and intake [28]. As an example of rumen dysfunction and dietary impacts on rumen motility, these fescue ergovaline effects on rumen function are of concern, as bacterial and fungal communities are important towards the animal’s response to fescue toxicosis [29,30,31]. As continued research advances the understanding of motility and digesta passage, their effects on the rumen microbiome and the potential to interact with the host via the ecological niches established by the rumen sacs will help to define further contributions to microbiome variation.

### 2.2. Ruminal Papillae Variation among the Different Sacs

Ruminal papillae are organs of absorption, extending from the rumen epithelium to optimize surface area for short-chain fatty acid (SCFA) absorption. Short-chain fatty acids account for over 70% of the metabolizable energy supply [17,32,33]. Papillae distribution, size, width, and volume are closely related to host feeding habits, forage availability, forage quality, particle size, and digestibility [17,32,33,34]. Papillae continuously adapt to changes in host diet and stratification of digesta that occur throughout cattle production, which enable its host to better absorb VFAs. In regions of the rumen with greater amounts of digesta, dense lawns of papillae exist, improving the ability to absorb VFAs. In regions such as the dorsal sac, there is a lower density of papillae, as VFA concentrations are reduced due to limited digestion and fermentation activity. As a result of these differences in biochemistry and physiology, papillae from various sacs differ in both size and shape [35]. Variations in papillae density have been observed because of increased proportions of propionate and butyrate produced by the fermentative ruminal bacteria [36]. These VFAs can increase ruminal blood flow, stimulating mucosal mitosis and epithelial proliferation, which increases the size and number of papillae, ultimately enhancing the reticulo-rumen’s ability to absorb VFAs [17,34,37]. Papillae VFA absorption contributes to maintaining rumen VFA concentrations and pH, which supports microbial fermentation of forage feedstuffs [38,39,40,41].

Ruminal papillae morphology and count are related to ruminant type. Ruminants can be classified based on the source of feed, which has evolutionarily impacted their anatomy and physiology. These categories include concentrate selectors, intermediate, and grass/roughage eaters [42]. Importantly, each ruminant type has varying distributions of papillae based on their diets. Concentrate selectors have a more even distribution of papillae seen throughout their rumen due to the increased passage rate and digestibility of their diet. Some concentrate selectors have papillae on the pillars within their rumen. Grass/roughage eaters or browsers, including cattle, have a characteristically uneven distribution of papillae throughout the rumen, reflecting the stratification of feed particles and thus, regional differences of microbial activity within their rumen [17]. Generally, within cattle, the rumen is devoid of papillae on the pillars and in the dorsal sac, depending on diet [17]. With significant differences in papillae configurations among ruminant types, it is consistently noted that the most numerous and extended papillae are located on the cranial and caudodorsal blind sac floor. The caudodorsal blind and cranial sac papillae retain greater surface enlargement factors (SEF = 2 × papillary surface + basal surface over the basal surface) due to increased VFA concentration [17].

The ventral sac papillae structure has been well-defined, due to ease of access and its dense lawn of papillae. However, the ventral sac is unlikely to be a complete representation of rumen papillae morphology and structure. Due to factors, such as digesta stratification, pH, and feedstuff digestibility, there may be distinct differences among the papillae in different rumen sacs. Micro- and macroscopic fluctuations in papillae morphology were observed through dry and lactation periods as an impact of increased dietary concentrates [39]. Twelve rumen-cannulated Holstein cows were sampled from the ventral, caudoventral blind, and caudodorsal blind sacs. There was an overall greater surface area of papillae with increased concentrates, indicating papillae respond to enhanced microbial breakdown of the easily fermentable organic matter through the increase in papillary growth rate [39]. Further differences were identified among the three different rumen sacs, with the ventral sac containing the greatest surface area of papillae. The caudodorsal blind sac also increased in papillae density, but the caudoventral blind sac did not have significant differences after concentrates were added [39]. Increases in papillae density provide greater surface area for absorption of VFA in the rumen. The caudoventral blind sac typically has a greater amount of liquid digesta due to the stratification of rumen contents. During acclimation to high-grain diets, the passage rate increases and therefore, more liquid digesta is cycled. However, the greater liquid accumulation does not deviate far from the average environment of the caudoventral blind sac, which may explain why no significance was determined.

Papillae express a variety of genes facilitating barrier defense, pH, receptors, and transporters for absorption of VFAs. Core genes expressed in papillae remain constant throughout a ruminant’s life due to their importance in host health. However, as mentioned previously, diet, location, and stress may impact ruminal epithelial tissue. In heat-stressed cattle (28 °C, temperature–humidity index = 76), lactating Holstein dairy cows had an increased expression of proteins involved in the AMP-activated protein kinase (AMPK) and insulin signaling pathways contrasted to thermoneutral cattle (15 °C, temperature–humidity index = 60). Expression of proteins was upregulated for those involved in the chaperone-mediated folding of proteins, whereas those involved in the antioxidant defense system were downregulated [43]. These changes in expression are important for host health, describing the gross physiological adaptations of ruminal papillae to heat stress. Yet, when diet is examined as a factor, no significant differences in epithelial gene expression were observed in cannulated Holstein cows, fed varying amounts of high-quality hay and decreasing percentages of concentrates [44]. However, evidence suggests that epithelial receptor expression may differ when different diets are fed, such as in Toll-like receptor genes. Increasing dietary starch increased expression of Toll-like receptor genes in goats which may be due to the genes’ ability to bind lipopolysaccharide and initiate a host immune response [44,45,46]. In a study conducted by Kern et al., steers with either high or low intake and high or low gain were examined for differentially expressed genes. There were differentially expressed genes determined in steers with high gain and low intake (HL), against the other efficiency type steers [47]. One gene upregulated in HH steers was lipopolysaccharide-binding protein (LBP), which aids in host defense and contributes to innate immune responses [47]. It was hypothesized that more efficient steers had less immune response which may drive improved production without wasting energy on inflammation and immune responses [47]. The gene Regulator of G Protein Signaling 5 (RGS5) was also differentially expressed in HL steers, where it was upregulated when measured against the HH steers [47]. In murine studies, RGS5 knockout mice were observed to have greater fat mass regardless of diet [48], which may also influence HL steers’ residual feed intake. RGS5 was also shown to be involved with arterial wall development in mice [47,49], which may facilitate the development of a more efficient rumen vasculature arrangement. Papillae are fixed to the rumen epithelium where they aid in absorption of VFAs, metabolic activity, and barrier protection, but they also support the epithelial microbial community. An epimural microbial community establishes throughout the entirety of the rumen given that they are attached to the epithelium or within the papillae that comprise every sac. These epimural microbes are distinct from the other microbial communities in that they inhabit the rumen epithelium, scavenge oxygen, recycle rumen epithelial tissue, and serve as an interface between the host and microbiome.

## 3. Microbial Community Variation, Fractions, and Importance in the Ruminant

Conditions of the rumen allow for a diverse community of organisms to grow and individual groups and species to develop complementary and syntrophic relationships [4]. The rumen consists of primarily anaerobic microbes, but facultative anaerobes inhabiting the epithelium are also present, which scavenge the minimal amounts of oxygen that enter through the esophagus [7]. There are consistently highly fermentable feedstuffs entering the rumen, allowing the ruminal environment to favor exceedingly active fermentation through microbial communities and thus, the breakdown of carbohydrates and proteins into energy for both the host and microbes.

### 3.1. Epimural Microbial Community

There are ranges of different ecological regions associated with various microbial communities throughout the rumen [4,50,51], including the liquid, solid, and epimural digesta. Collection and observation of each digesta type allow researchers to better understand different communities and how they interact. Numerous studies have focused on the fiber-adherent and planktonic communities [52,53,54,55], but the epimural community, or the microbes residing on the rumen epithelium, has been far less investigated. The minimal investigations conducted on the epimural community may be due to the lesser abundance of these microbes in relation to the whole microbial population and due to the difficulty of sampling. However, there are many vital responsibilities of epimural microbes, including oxygen scavenging, epithelial tissue recycling, metabolizing amino acids, and hydrolyzing urea [14,15,56,57,58,59], and they serve as the interface between the host and the luminal microbes. Therefore, further research is necessary to fully grasp the extent of epimural microbial capabilities.

In dairy cattle, researchers have noted impactful changes to the rumen microbiome when cows are often gradually transitioned from a silage- and concentrate-based ration. One such study focused on the ruminal planktonic, fiber-adherent, and epimural archaea and bacteria and their variation attributed to the transition from a silage- and concentrate-based ration to pasture [60]. The epimural microbial community was much less diverse contrasted to the other fractions, which has been verified by other studies [52,61]. The reduced diversity was likely attributed to the increased spatial environment and different degradative functions required for substrate digestion among the strains of the other microbial fractions compared to those that reside along the rumen epithelium. Importantly, the authors found that, as opposed to current hypotheses on epimural microbial stability throughout dietary changes [62], the epimural communities were significantly influenced by the dietary change. This observation has been supported by Petri et al. (2013), where researchers found significant variability among the rumen epimural population during the transition from a forage to a high-concentrate diet, during acidosis, and after recovery [61]. Among most of the studies discussed, the researchers noted the rumen still had a core set of variable microbes among all planktonic, fiber-adherent, and epimural archaea and bacteria [60,61]. These data demonstrate the importance of the epimural microbial community to dietary and nutritional variation.

A recent study focused on the epimural community among the cranial, ventral, caudodorsal blind, and caudoventral blind rumen sacs in Holstein cattle, which highlighted variation in community structure and composition as a function of sample location indicating intra-ruminal microbial variation [3]. Most variation observed among the rumen sacs sampled occurred among the caudodorsal blind sac (CDBS), caudoventral blind sac (CVBS), and ventral sac (VS) [3]. Specifically, the CDBS bacterial evenness and richness, as measured by Shannon’s diversity, were greater than that of the CVBS. The CDBS also displayed the greatest degree of separation using Bray–Curtis dissimilarity analyses and was significantly different from the VS and CVBS communities [3]. These distinctions in microbial structure among the CDBS and the two ventrally located sacs can further be explained due to stratification and rumen motility. Movement through the rumen is expected to affect the dorsal and medial locations, which will generate increased passage of feedstuffs through the CDBS. This may provide an explanation as to why greater relative abundance and diversity differences were observed between the CDBS and the two ventral locations in this study. This study strengthens the concept of multiple rumen sacs and digesta-type sampling to obtain an accurate assessment of the complete rumen microbiome.

### 3.2. Interactions among the Fiber-Adherent and Planktonic Microbial Communities

Stratification of feedstuffs, anatomical variation, and constant contractions generate population distinctions in ruminal digesta levels, causing a natural divergence between fiber-adherent and planktonic communities. Contractions within the rumen primarily depend on particle size, intake, and digestibility of the feedstuff, where larger particles with low specific gravity will settle in the fiber mat, separating the gas and liquid levels [4]. The fiber-adherent community, consisting of predominantly cellulolytic bacterial species, attaches to solid feed particles in the fiber mat to break them down. There is consistent fermentation and breakdown of these solid feed particles attributing to the fiber-adherent community maintaining the greater percentage of the bacterial biomass in the rumen [3,4]. Forage particles that are too large will return to the mouth via rumination to be re-masticated and increase the surface area for fiber-adherent microbes to attach and break these particles down. The liquid-associated or planktonic microbial community regularly obtains a variety of feedstuffs due to the constant redistribution of feedstuffs around the rumen. Planktonic microbes take advantage of soluble carbohydrates floating in the rumen liquid to produce various VFAs.

Stratification throughout the rumen promotes microbial community diversity among the five rumen sacs [7,10]. For example, the ventral and caudoventral blind sacs are composed of mostly liquid digesta, which leads to the assumption that primarily the planktonic community will inhabit these rumen sacs. The upper portion of the rumen, including the caudodorsal blind and dorsal sacs, retains little amounts of liquid and mostly consists of gas and solid feedstuffs [10]. Presumably, in this case, predominantly epimural and fiber-adherent communities would reside in these sacs. However, with contractions shifting liquid digesta around the rumen, the opportunity exists for planktonic microbes to be identified in the dorsal and caudodorsal blind sacs [63]. Microbial communities among different ruminal niche environments fluctuate based on how they interact with the host and where they reside during the production changes to cattle [3,43,63]. Thus, it would be advantageous to improve our understanding of these microbial deviations that occur in normal cattle production causing ruminal disorders and decreased efficiency in cattle production to enhance our ability to relieve these disorders.

Many studies have focused on identifying precise differences between the fiber-adherent and planktonic microbial communities. However, it is unclear whether these distinctions are due to bacterial and methanogen community, based on diet changes, or transition periods for dairy cattle. Bacterial microbial dynamics were characterized during four daily feedings of two cannulated Holstein cows utilizing Automated Ribosomal Intergenic Spacer Analysis (ARISA) which can detect a deeper degree of the bacterial variation [64]. Solid and liquid digesta samples were removed from the ventral sac at six different time points during a single feeding cycle. Notable contrasts between the solid and liquid fraction bacterial communities were observed. However, the liquid community generally had greater changes during the feeding cycles [64]. Decreased bacterial community shifts in the solid digesta may be due to overall less movement throughout the rumen in this level. Contractions provide greater passage for liquid digesta and thus the liquid-associated community may be mixing with other microbial species, causing increased fluctuations in this community. Bacterial and methanogen communities were investigated from all three digesta levels through cannulated dairy cattle, which highlighted differences in the abundance of taxonomic groups in the liquid- and solid-associated communities [65]. Although no significant differences among the solid and liquid communities were measured, there were significant differences in the relative abundance of the major bacterial phyla in the rumen, such as Firmicutes, Bacteroidetes, and Proteobacteria [65]. Consistent movements due to the primary contraction cycle may be further integrating the solid and liquid bacterial communities, yet differences were observed due to abundances between communities. There are well-defined structural differences as well as environmental and chemical differences among the solid and liquid microbial communities. Both interactions and dissimilarities exist among solid and liquid communities, further warranting the necessity to sample from both communities to attain the most comprehensive samples of the rumen. There are a multitude of unavoidable factors contributing to the fluctuations within the ruminal environment such as diet and location changes when progressing through the beef production cycle. These changes can cause production losses due to health concerns such as acidosis, therefore further investigation into how these changes affect the microbial communities and how to best alleviate the consequences that may transpire is critical.

## 4. Conclusions

Ruminants are foregut fermenters that rely on microbial communities to break down feedstuffs into energy through fermentation [66,67]. While the rumen may be viewed as simply a large fermentation vat, it is much more complex than that where distinct anatomical musculature demarcates the rumen into five distinct rumen sacs. Each of these five rumen sacs generates diverse ecological niches due to different ruminal factors, such as stratification, contractions, and passage rate. The fiber-adherent, planktonic, and epimural microbial communities are able to inhabit specific environments, such as the solid feed particles, liquid digesta, or rumen epithelium, to thrive and outcompete other species. With variation both anatomically and microbially within the rumen, it is critical to further explore the variation among the five rumen sacs and their respective microbial communities and digesta fractions. This would provide support for the need to sample from multiple ruminal locations to better understand the microbial interactions within the rumen. In addition, the microbes identified in these ruminal niche environments may help further define the complex interactions occurring between the rumen microbiome and its host. Continuing research into the complex ruminal environment may lead to discoveries on how the five sacs and the microbiome can impact the host’s feed efficiency, nutrition, and health during the segmented beef production cycle.

## Data Availability

Data sharing is not applicable, as no new data were created or analyzed in this review article.
